# Protein Nanomachines

**DOI:** 10.1371/journal.pbio.0020073

**Published:** 2004-03-16

**Authors:** Michael Strong

## Abstract

At the interface of biology and nanotechnology lies an area of research that aims to construct molecular-scale machines based on protein and nucleic acid

In 1959 Richard Feynman delivered what many consider the first lecture on nanotechnology. This lecture, presented to the American Physical Society at the California Institute of Technology, prompted intense discussion about the possibilities, or impossibilities, of manipulating materials at the molecular level. Although at the time of his presentation, the manipulation of single molecules and single atoms seemed improbable, if not impossible, Feynman challenged his audience to consider a new field of physics, one in which individual molecules and atoms would be manipulated and controlled at the molecular level (Feynman 1960).

As an example of highly successful machines at the “small scale,” Feynman prompted his audience to consider the inherent properties of biological cells. He colorfully noted that although cells are “very tiny,” they are “very active, they manufacture various substances, they walk around, they wiggle, and they do all kinds of wonderful things on a very small scale” (Feynman 1960). Of course, many of these “wonderful things” that he was referring to are a result of the activities of proteins and protein complexes within each cell.

The field of nanotechnology has indeed emerged and blossomed since Feynman's 1959 lecture, and scientists from many disciplines are now taking a careful look at the protein “machines” that power biological cells (Drexler 1986). These “machines” are inherently nanoscale, ranging in width from a few nanometers (nm) to over 20 nm, and have been carefully refined by millions of years of evolution.

As a graduate student in molecular biology, I have been especially interested in creative approaches to bridging the fields of biology and nanotechnology. Both DNA and protein molecules possess a number of intrinsic characteristics that make them excellent candidates for the assembly of dynamic nanostructures and nanodevices. Properties such as the site-specific molecular recognition among interacting protein molecules, the template-directed self assembly of complementary DNA strands, and the mechanical properties of certain protein complexes have enabled bionanotechnologists to envision a molecular world built “from the bottom up” using biological-based starting materials.

In my own research, I have been very interested in investigating protein interactions and protein pathways on a genome-wide scale. In many ways, protein pathways are analogous to nanoscale “assembly lines,” since protein pathways often involve a series of proteins that act in successive order to yield a particular molecular “product” or perform a particular molecular function. While these protein-based “assembly lines” are commonplace within biological cells, they prompt two interesting questions with respect to the field of nanotechnology. First, can we mimic these multicomponent protein-based “assembly lines” on nanofabricated surfaces? And, second, can we tailor these “nanoscale assembly lines” to perform new and unique tasks?

Nanomechanical protein complexes, such as the rotary ATP synthase complex, have also generated much interest from a nanotechnology standpoint (Soong et al. 2000). These protein complexes enable highly controlled mechanical motion at the nanoscale and may some day lead to novel rotary machines that function as molecular motors for a variety of nanoscale applications.

In order to fully exploit these nanoscale protein machines, it is of prime importance to be able to position individual proteins and protein complexes at the nanoscale.

Progress in this area has recently been reported by Yan et al. (2003), who developed a method to construct two-dimensional protein arrays using DNA-directed templates. Building on work pioneered by Nadrian Seeman (Seeman 2003), Yan et al. constructed two-dimensional DNA “nanogrids” by exploiting the pairing that occurs between complementary DNA strands (Figure 1). The two-dimensional DNA nanogrid exhibits a repeating periodic structure (Figure 1B) due to the inherent qualities of the individual DNA tiles that make up the nanogrid (Figure 1A). The distance between adjacent tile centers is approximately 19 nm (approximately 4.5 turns of the DNA double helix plus the diameter of two DNA helices).

**Figure 1:**
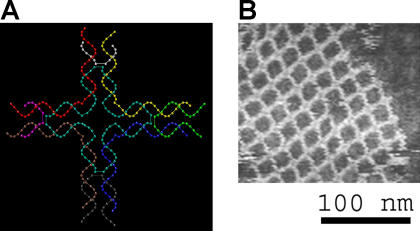
Self-Assembled DNA Nanostructures (A) DNA “tile” structure consisting of four branched junctions oriented at 90° intervals. These tiles serve as the primary “building block” for the assembly of the DNA nanogrids shown in (B). Each tile consists of nine DNA oligonucleotides as shown. (B) An atomic force microscope image of a self-assembled DNA nanogrid. Individual DNA tiles self-assemble into a highly ordered periodic two-dimensional DNA nanogrid. (Images were kindly provided by Thomas H. LaBean and Hao Yan.)

Yan et al. utilized these DNA nanogrids to assemble periodic protein nanoarrays. The DNA nanogrid, in this case, served as a molecular scaffold for the self assembly of protein molecules into ordered arrays. In order to control the location of protein assembly, Yan et al. first tethered a covalently linked biotin moiety to the central region of each DNA tile. The biotin was covalently linked to one of the DNA strands at the position corresponding to the center of the tile. This design resulted in a uniform array of biotinylated tiles, with each biotin moiety separated by about 19 nm. The authors then added streptavidin, a protein that has a strong binding affinity for biotin, to form a periodic streptavidin protein array on top of the biotinylated DNA lattice. The resulting array represents the first periodic, self-assembled DNA lattice in which individual protein molecules are precisely positioned into a periodic array with nanometer dimensions.

It is interesting to consider some of the applications of self-assembled protein arrays. Soong et al. (2000) demonstrated that the ATP synthase protein complex could be used to power the rotation of an inorganic nickel “nanopropeller.” ATP synthase is a multisubunit protein complex with a domain that rotates about its membrane-bound axis during the natural hydrolysis of ATP within a cell. Soong et al. attached a nanoscale inorganic “propeller” to the rotary stalk of ATP synthase, creating a “rotary biomolecular motor.” It is intriguing to consider the construction of an ordered array of ATP synthase driven nanomachines, each positioned precisely along a DNA scaffold, similar to that described by Yan et al. Such an assembly, combined with proposed “nanogears” (Han et al. 1997), may one day enable the construction of nanoscale variations of the traditional “gear-train” and “rack-and-pinion” gearing systems. Construction of such systems may facilitate the design of machines that can transmit and transform rotary motion at the nanoscale.

In addition to rotary biomolecular motors, proteins that undergo substantial conformational changes in response to external stimuli might also find some interesting uses in nanoarrays. Dubey et al. (2003) are working on methods to exploit the pH dependent conformational changes of the hemagglutinin (HA) viral protein to construct what they term viral protein linear (VPL) motors. Proteins that undergo substantial conformational changes in response to environmental stimuli may facilitate the design of nanoscale machines that produce linear motion (Drexler 1981), as opposed to rotary motion. At neutral pH, the HA_2_ polypeptide forms a compact structure composed of two α-helices folded back onto each other. At low pH, HA_2_ undergoes a substantial conformational change, which results in a single “extended” helix. This conformational change results in a linear mechanical motion, with a linear movement of approximately 10 nm (Dubey et al. 2003). It would be interesting to investigate the applications of ordered arrays of dynamic VPL motors, since an array of such “hinge” structures may enable the coordinated linear movement of hundreds of tethered macromolecules in a synchronous manner.

The work of Yan et al. (2003) has opened up exciting new avenues in the field of nanotechnology and has provided the molecular framework for the construction of dynamic protein-based assemblies. It is foreseeable that variations of these same DNA scaffolds will eventually be used for the design and construction of more complex protein-based assemblies, such as nanoscale “assembly lines” or periodic arrays of dynamic motor proteins. This work is important to me because it demonstrates not only that it is possible to create uniform arrays of protein biomolecules using biomolecular scaffolds, but the study also emphasizes the important role that molecular biology will undoubtedly play as the field of nanotechnology matures.

As the field of nanotechnology continues to evolve, it is likely that we will see many more nanotechnology applications utilizing biological macromolecules. Toward the end of Richard Feynman's 1959 lecture, he quipped, “What are the possibilities of small but movable machines? They may or may not be useful, but they surely would be fun to make.”

## Abbreviations

HA: haemagglutinin
nm: nanometer
VPL: viral protein linear
